# Selecting the right gate to identify relevant cells for your assay: a study of thioglycollate-elicited peritoneal exudate cells in mice

**DOI:** 10.1186/s13104-017-3019-5

**Published:** 2017-12-06

**Authors:** Micely D. R. Hermida, Rafaela Malta, Marcos D. P. C. de S. Santos, Washington L. C. dos-Santos

**Affiliations:** Laboratório de Patologia e Biointervenção, Fundação Oswaldo Cruz, FIOCRUZ-BA, Instituto Gonçalo Moniz, Rua Waldemar Falcão, nº 121, Candeal, Salvador, Bahia CEP 40296-710 Brazil

**Keywords:** Flow cytometry, Peritoneal exudate cells, Thioglycollate stimuli, Cell size and granularity

## Abstract

**Objective:**

In this study, we investigate the diversity and modulation of leukocyte populations represented in the gates defined by size and granularity at different time points of thioglycollate-induced peritonitis in mouse.

**Results:**

The inflammatory cells were distributed into four regions (R1–R4) of a data plot graph defined by cell size and granularity. R1 and R2 contained agranular cells that were small in size and predominately included T (CD3^+^) lymphocytes along with B (B220^+^) lymphocytes. Macrophages (F4/80^+^) were the predominant cells found in the R3 region. However, these cells were present in all regions, albeit at a lower frequency in R1 and R2. Granulocytes (Gr1^+^) were mainly distributed in R3 and R4. The wide distribution of F4/80^+^ and Gr1^+^ cells may reflect the recruitment and activation state of the different macrophage and granulocyte populations. Based on these observations, size and granularity may contribute to an initial step in the analysis and sorting of thioglycollate-elicited peritoneal exudate cells. However, the developmental stage and cell activation state may interfere with cell segregation using size and granularity as parameters.

**Electronic supplementary material:**

The online version of this article (10.1186/s13104-017-3019-5) contains supplementary material, which is available to authorized users.

## Introduction

The experimental induction of peritonitis in mice with thioglycollate (TGM) allows a variety of leukocytes to be obtained in large numbers under sterile conditions that are suitable for in vitro cultivation and a variety of experiments [1–3]. For example, neutrophils, macrophages and lymphocytes may predominate during different stages of TGM-induced peritonitis [3, 4]. In many studies, cell size and granularity alone or in combination with antibody labeling are used for the analysis and sorting of relevant leukocyte populations by flow cytometry. The distribution of TGM-elicited PECs when plotted by cell size and granularity results in the visualization of at least four distinct regions. Although these regions predominately correspond with lymphocytes, macrophages and polymorfonuclear leukocytes, the cell composition is diverse and further enhanced by peritonitis progression [4]. In this work, we use morphology together with immunophenotyping to characterize the TGM-elicited PECs distributed in the most representative clusters defined by size and granularity on flow-cytometric dot plots. The aim of this work is to minimize misinterpretation of cell analysis data by providing a strategy that takes advantage of the cell diversity during the course of peritonitis.

## Main text

### Methods

#### Kinetics of inflammatory cell influx into the peritoneal cavity in thioglycollate-induced peritonitis

Peritonitis was induced in BALB/c mice, 6- to 8-week-old of both sex, by injecting 3 ml of a sterile 3% (wt/vol) thioglycollate (catalog # T9032, Sigma Aldrich, USA) solution. PECs were collected after 4, 8 and 12 h and after 1, 2, 4, 10, 20, 40 and 100 days by washing the peritoneal cavity twice with cold Ca^2+^ and Mg^2+^-free Hanks’ balanced salt solution (HBSS; Sigma Aldrich, USA) containing 20 IU/ml heparin. The number of cells collected from each animal was estimated using a Neubauer chamber. Cell viability was assessed by trypan blue dye exclusion, and cell populations were defined by morphology using cytospin preparations and specific antibodies for detection by flow cytometry. All the experiments were independently repeated twice using three animals in each group.

#### Flow cytometry analysis

The cells (1–2  ×  10^6^/stain) were stained with the following fluorescein isothiocyanate-conjugated antibodies: anti-CD3e (145-2C11, catalog# 553061), anti-B220 (RA3-6B2, catalog# 553087) and anti-GR-1 (RB6-8C5, catalog# 553126) (BD-Bioscience; USA) and phycoerythrin (PE)-conjugated antibody was anti-F4/80 antigen (BM8.1, catalog# FP20066010; Caltag, USA) as previously described [1]. Unlabeled or isotype-matched stained cells were used as controls. The cells were analyzed using a FACSAria III flow cytometer and FlowJo Software (Tree Star, USA).

#### Cell sorter and leukocyte morphology

PECs were sorted based on their size and granularity using a FACSAria III sorter (BD Biosciences, USA), cytocentrifuged at 500 rpm onto glass slides and stained with Papanicolaou’s, hematoxylin and eosin, or the Luna’s (to detect eosinophil granules) stain techniques [5–7]. Leukocyte were identified using previously defined characteristics [8].

### Results

#### Kinetics of leukocyte recruitment in thioglycollate-induced inflammation

**Fig. 1:**
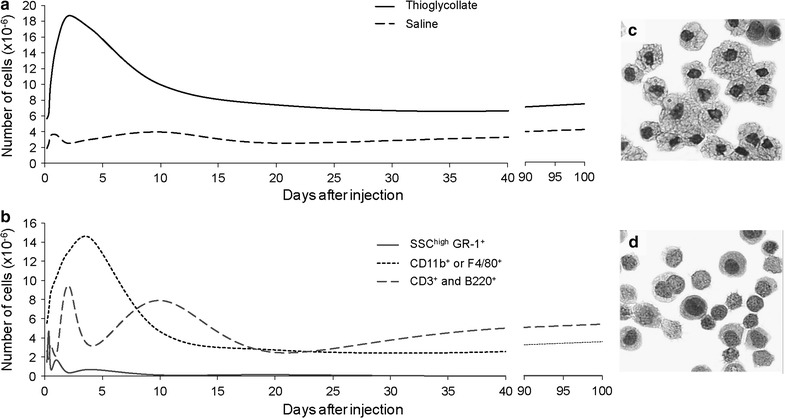
Kinetics of leukocyte recruitment into the peritoneal cavity and phenotypes after thioglycollate stimulation. **a** Total number of cells in animals injected with thioglycollate (solid line) or saline (dashed line). **b** The percentage of each cell type recruited to the peritoneal cavity; including neutrophils (solid line), monocyte/macrophages (dotted line) and lymphocytes (dashed line) cellular phenotypes. Images of PECs collected 4 days after the injection of **c** thioglycollate or **d** saline stained following the Papanicolau technique

The number of cells present in the peritoneal cavity of animals injected with thioglycollate rose from 5.7 ± 2.1 × 10^6^ at 4 h to 1.5 ± 0.7 × 10^7^ on the 1st day and to 1.9 ± 1.0 × 10^7^ on the 2nd day after thioglycollate injection. After the second day, the number of cells in the peritoneal exudate slowly decreased and plateaued at approximately 9 × 10^6^ cells between the 25th and the 100th day after stimulation. The number of leukocytes in the peritoneum of animals injected with saline rose from 1.9 ± 1.0 × 10^6^ at 4 h to 3.6 ± 0.8 × 10^6^ at 24 h and declined to basal levels 20 days after saline injection (Fig. 1a). Mononuclear phagocytes (CD11b^+^ or F4/80^+^) predominately influence the shape of the peritoneal cell recruitment curve through the 7th day of inflammation. Neutrophil (SSC^high^ GR-1^+^) recruitment reaches a small peak on the first day and lymphocytes (CD3^+^ and B220^+^) become the predominant recruited cell after the 7th day of inflammation (Fig. 1b).

#### Identification of total cell populations of the peritoneal inflammatory exudate based on their size and granularity

**Fig. 2:**
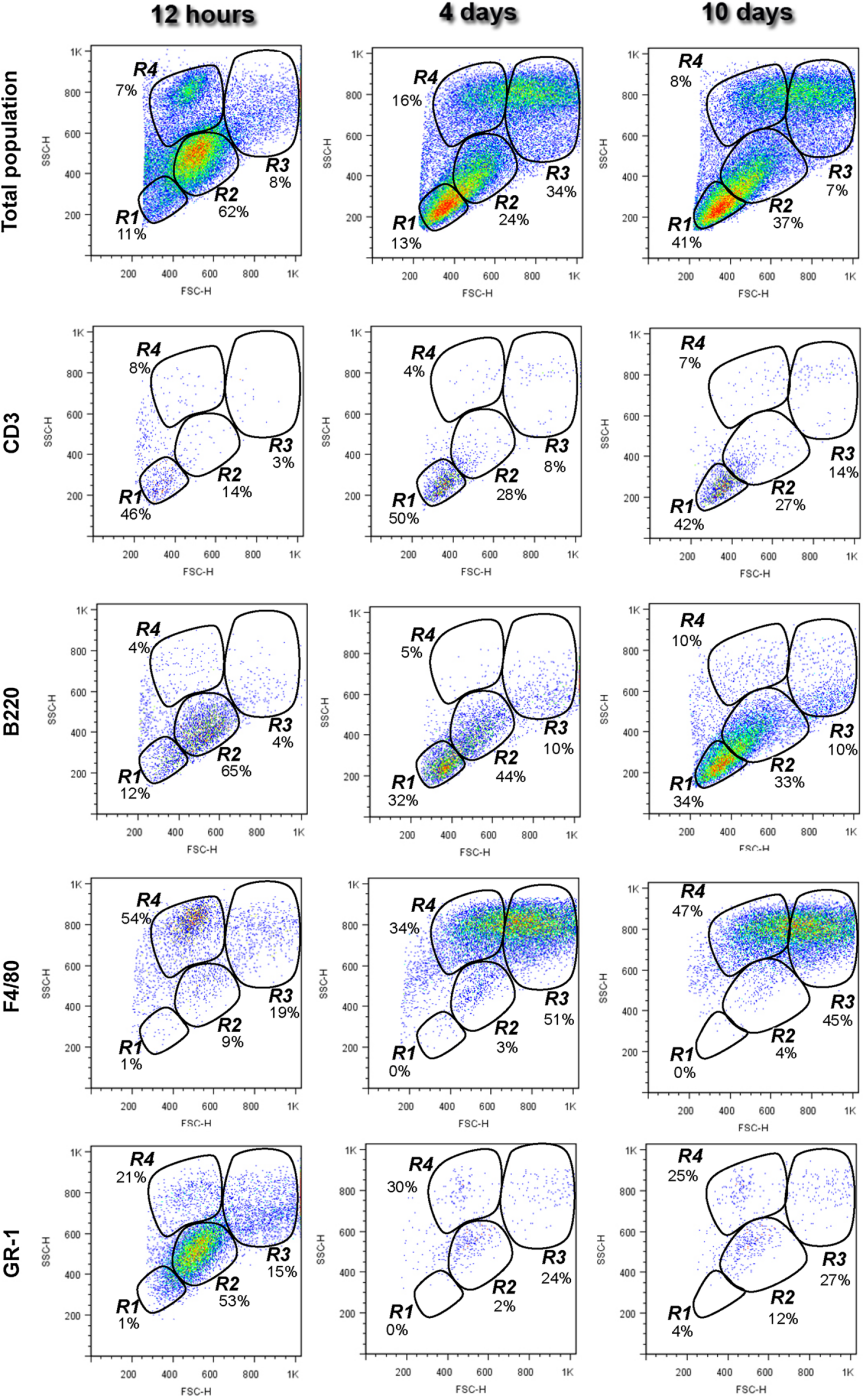
Inflammatory peritoneal cells contain a variety of immune cell subsets at different time points following thioglycollate stimulation. Flow cytometry and morphological analysis of thioglycollate-elicited peritoneal exudate cells (PEC) after 12 h, 4 days and 10 days. The gating strategy based on the cell size and granularity (forward/side scatter profile) defined four regions (R1–R4). Scattering of immune inflammatory cells based on surface molecules and size and granularity

The plotting of total PEC distribution by size and granularity resulted in the visualization of four distinct regions, confirming previous data by Cook and colleagues [3]. R1 and R2 represent small cells with low granularity, R3 represents cells that are large size with intermediate granularity and R4 represents cells intermediate in size with high granularity (Fig. 2). The cells in R1 (8 ± 3%) and R2 (55 ± 9%) predominated 12 h after TGM stimulation. The distribution pattern changed by the 4th day of peritonitis, with a fourfold increase in the proportion of the cells represented in the R3 (36 ± 8%) region, a small increase in the proportion of the cells represented in the R1 (17 ± 5%) and R4 (13 ± 8%) regions and a relative decrease in the proportion of cells in the R2 (24 ± 0%) region. On the 10th day of peritonitis, the cell distribution became more evenly distributed between the R1, R2 and R3 regions and the R4 region returned to the proportion observed at 12 h.

#### Distribution of lymphoid and myeloid leukocytes among the R1–R4 regions at different time points after TGM peritonitis induction

The distribution of lymphoid and myeloid cells in the R1–R4 regions is summarized in Fig. 2 and in Additional file 1: Table S1. As expected, after 12 h of stimulation, most of the lymphocytes (CD3^+^ and B220^+^ cells) were distributed in regions R1 and R2. B220^+^ cells (B lymphocytes) predominated in R2 (52 ± 12 to 21 ± 12%), whereas CD3^+^ cells (T lymphocyte) were more evenly distributed between R1 and R2. Furthermore, a considerable number of B and T lymphocytes were observed in the other regions (R3 and R4). Cells expressing F4/80 and GR1, which are more often associated with myeloid cells (macrophages and granulocytes), were widely distributed in regions R2–R4. F4/80^+^ cells, which include various macrophage populations, were mostly represented in the R3 (60 ± 12%) region 4 days after stimulation. A high proportion of these cells were observed in R2 (18 ± 13%) and R4 (47 ± 11%) 12 h after peritonitis induction. However, as a whole, the R4 region contained only (13 ± 9%) of the PECs. The proportion of F4/80^+^ leukocytes was decreased in R2 after 4 (2 ± 3%) and 10 (8 ± 6%) days of peritonitis. The pattern of the Gr1^+^ cell distribution was more diverse, with an increased proportion of the cells in R2 (59 ± 8%) after 12 h of peritonitis, a shift to the R3 (30 ± 8%) and R4 (36 ± 8%) regions after 4 days of peritonitis, followed by a more even distribution among the R2–R4 regions after 10 days of TGM peritonitis. This distribution of F4/80^+^ and Gr1^+^ cells may reflect the recruitment and activation state of the different macrophage and granulocyte populations.

#### Morphologic characterization of the cell subpopulations present in each region (R1–R4)

**Fig. 3:**
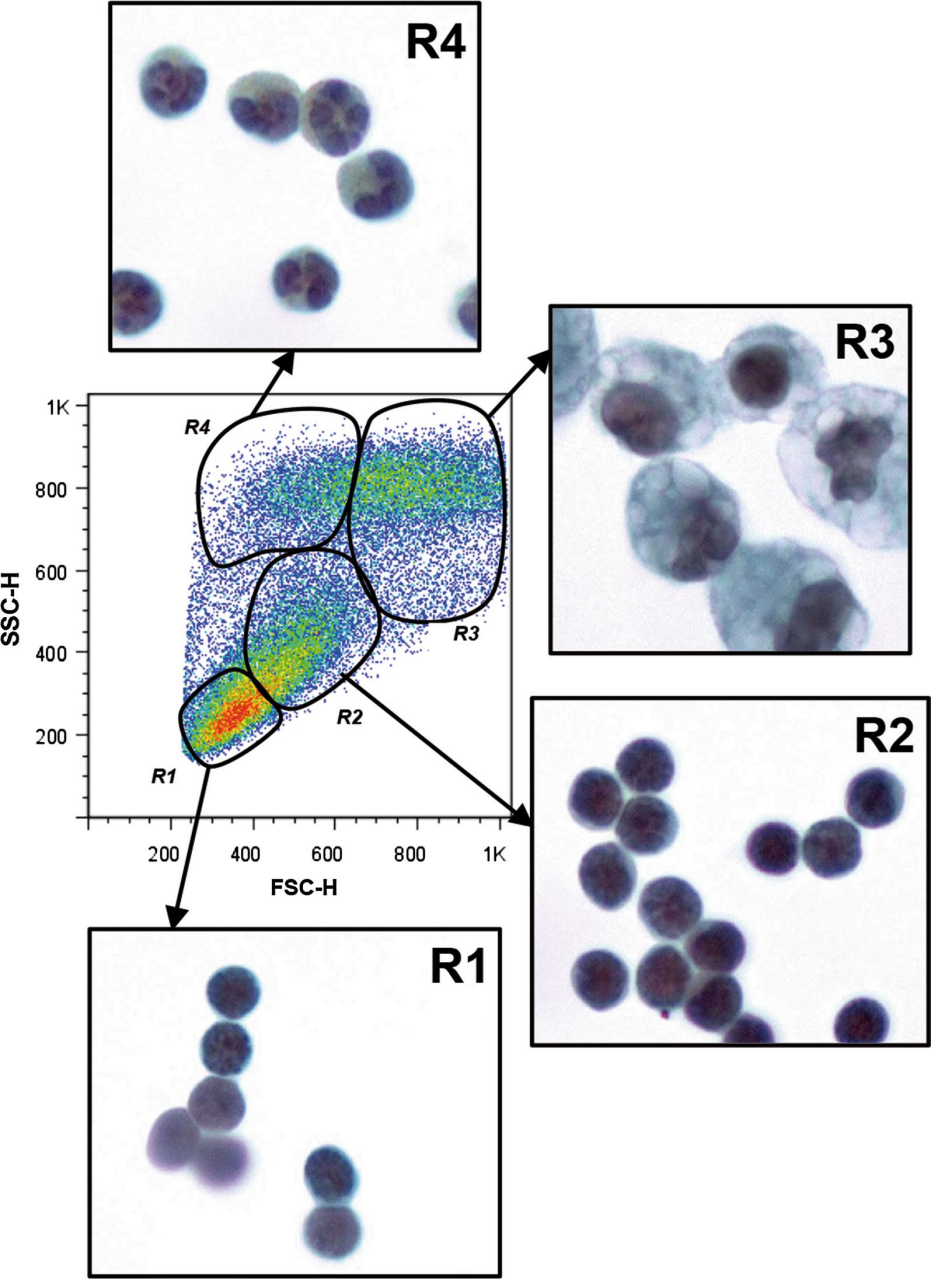
Morphological characterization of inflammatory peritoneal cells 4 days after stimulation in each region (R1–R4). Inflammatory PECs from different regions (R1–R4) were FACS-sorted and their morphological characteristics were defined. PECs were stained using Papanicolaou’s stain technique

Cells present in R1 and R2 predominantly exhibited lymphocyte morphology (Fig. 3). The leukocyte counts showed that 92 ± 3% of lymphocytes were found in R1 and 94 ± 3% were found in R2 (Additional file 1: Table S2). Most of the cells in R3 (97 ± 1%) displayed macrophage morphology, whereas R4 predominantly contained cells with polymorphonuclear (94 ± 3%) morphology (Fig. 3). Most of the polymorphonuclear cells present in R4 contained deeply red stained granules indicative of eosinophils as determined by the Luna staining technique (Additional file 2). Additionally, some cells with eosinophil morphology (large cytoplasm and ring-shaped nucleus) expressed the F4/80 antigen (Additional file 3). Cells with macrophage/monocyte morphology were sorted from all regions, though these cells were much less prevalent in regions R1 and R2.

### Discussion

Thioglycollate-induced peritonitis reproduces the aspects of a complex chronic infection with a sequential change in cell population and cell maturation. The initial influx of neutrophils is followed by monocytes, which become the main cell population between day 3 and 6 of peritonitis. Subsequently, lymphocytes predominate, the timing of which coincides with the expression of addressins by endothelial cells [9]. The changes in cell populations and differentiation stages are reflected when cells are plotted by size and granularity as evident by the composition and size of the four regions presented in the graphs. For instance, the proportion of CD3^+^ and B220^+^ lymphocytes represented in R1 substantially increases on day 4 of peritonitis concomitant with the large increase in the absolute number of cells in the peritoneal cavity. Additionally, overlapping areas between clusters appear during the course of peritonitis. For instance, R1 and R2 are only individualized during the 4th day of peritonitis, whereas R3 and R4 are only individualized during the early stage of the response to the stimuli. Large numbers of F4/80^+^ GR1^+^ cells that are small in size, possibly resting macrophages, are contained within the R2 region along with predominant B lymphocytes. In contrast, R1 is more stably constituted by small lymphocytes. Changes in the composition of these clusters may also explain the differences in the definition of the regions related to these cell clusters in different studies [3, 4, 10]. Nevertheless, attention must be given to the variation in the phenotypes of the cells observed in the R1–R4 regions at different stages of TGM-induced peritonitis and the potential interference of this variety of cell populations in the proposed experiments.

As shown in Additional file 1, B lymphocytes were evenly distributed between R1 and R2. In fact, these cells comprised two subpopulations of B lymphocytes corresponding to B1 and B2. This finding was consistent with the description given by Cook and colleagues [3], who showed that the B1 lymphocytes represented in R2 were mixed with other cells such as small macrophages [3]. As shown in this study, these reputed B2 lymphocytes were found in R1 together with T lymphocytes [3]. Furthermore, some B lymphocytes were dispersed in different regions and might be overlooked during analysis and sorting studies using the common strategy of segregation by size and granularity followed by antibody labeling. A similar observation was made for other cell populations, such as macrophages. For instance, although microscopy analysis of R3 cells readily identified large macrophages containing cytoplasmic vacuoles associated with TGM peritonitis [3, 11], small macrophages were present in both this region and R2, where they were barely distinguishable from lymphocytes. Additionally, the R4 region is predominately composed of GR-1^+^ cells, some of which expressed the F4/80 antigen. Although F4/80 is generally considered to be a macrophage marker [12–14], other authors have reported that F4/80 is not exclusive to monocyte/macrophage populations [3, 15]. Indeed, other cells, such as eosinophils [16] and skin Langerhans cells [17], are also labeled by this antibody. Sorting and cytological analysis of this cell population using Luna and H&E staining confirmed that all of the eosinophils present in the PECs were segregated in R4 (Fig. 3; Additional file 2, Additional file 3).

Taken together, the data presented here show that the different phases of TGM-induced peritonitis represent an important source of a variety of leukocyte populations that are suitable for diverse experiments in pathology and inflammation. Using a combination of size and granularity analysis together with morphological analysis may contribute to a more rational use of antibodies for the identification and selection of relevant cell populations from PECs. This approach may help to decrease the arbitrariness involved in the selection of leukocyte populations in analytical and cell sorting studies.

### Limitations

In the study design, was without control group (non inflammatory cells). Because, it was difficult to sort cells from steady state animals. It would take many animals to obtain sufficient numbers of cells to perform the sorter.

## Additional files

**Additional file 1: Table S1.** Phenotypic analysis of distinct cell regions (R1–R4) sorted based on size and granularity in the peritoneal cavity at different time points after thioglycollate stimulation. **Table** **S2.** Morphological analysis of distinct cell regions (R1–R4) based on size and granularity in the peritoneal cavity 4 days after thioglycollate stimulation.

**Additional file 2.** Sorted cells in the R4 region have eosinophilic cytoplasmic granules. Cells stained with (A) H&E, (B) the Luna method, or (C) the Papanicolau technique.

**Additional file 3.** Cell immunophenotyping: To identify the neutrophils expressed F4/80 antigens, R4 cell from inflammatory PEC were sorted and cytocentrifugated at 500 rpm onto glass slides and fixed in cold acetone and subjected to immunolabeling. The slides were incubated with phosphate buffer saline (PBS) containing 1% bovine serum albumin (BSA), 10% normal goat serum, to block non-specific interactions. Purified F4/80 antigen antibody (Cl:A3-1, catalog # MCAP497, Serotec) was used at 5 µg/ml and incubated for 1 h at room temperature. After, wash the slides with PBS 1× and 0.05% tween 20 and incubated with secondary antibody anti-Rat IgG FITC (catalog # STAR69, Serotec) for 45 min in room temperature. For lobulated nucleus neutrophil identification, the slides were stain with DAPI.

## Abbreviations

PECs: peritoneal exudate cells
HBSS: Hanks’ balanced salt solution
FBS: fetal bovine serum
RPMI: Roswell Park Memorial Institute
PBS: phosphate-buffered saline
