# Supplementary material for: Selecting the right gate to identify relevant cells for your assay: a study of thioglycollate-elicited peritoneal exudate cells in mice
Source: BMC Res Notes. 2017 Dec 6;10:695. doi: 10.1186/s13104-017-3019-5 (PMC5718147; doi:10.1186/s13104-017-3019-5)
Supplement: Supplementary file 1 — Additional file 1: Table S1. Phenotypic analysis of distinct cell regions (R1–R4) sorted based on size and granularity in the peritoneal cavity at different time points after thioglycollate stimulation. Table S2. Morphological analysis of distinct cell regions (R1–R4) based on size and granularity in the peritoneal cavity 4 days after thioglycollate stimulation. [file 13104_2017_3019_MOESM1_ESM.doc]

| Table S1. Phenotypic analysis of distinct cell regions (R1-R4) sorted based on size and granularity in the peritoneal cavity at different time points after thioglycollate stimulation.   |  | | | | | | --- | --- | --- | --- | --- | | Cell subtype | | | | | | R4Regions (%) | R1 | | | | |  |  |  |  | | 7 ± 213 ± 9Total population | | | | | | CD38 ± 355 ± 9 | 35 ± 12 | 34 ± 28 | 7 ± 4 | 5 ± 2 | | B220 | 21 ± 12 | 52 ± 12 | 8 ± 4 | 4 ± 0 | | F4/80 | 2 ± 2 | 18 ± 13 | 17 ± 3 | 47 ± 11 | | GR-1 | 5 ± 6 | 59 ± 8 | 13 ± 3 | 14 ± 9 | |  |  |  |  |  | | 4 days |  |  |  |  | | Total population | 17 ± 5 | 24 ± 0 | 36 ± 8 | 13 ± 8 | | CD3 | 51 ± 0 | 25 ± 4 | 9 ± 2 | 3 ± 1 | | B220 | 34 ± 2 | 41 ± 4 | 12 ± 3 | 4 ± 2 | | F4/80 | 0 ± 0 | 2 ± 3 | 60 ± 12 | 25 ± 11 | | GR-1 | 0 ± 1 | 3 ± 2 | 30 ± 8 | 36 ± 8 | |  |  |  |  |  | | 10 days |  |  |  |  | | 11 ± 67 ± 2Total population | | | | | | CD337 ± 639 ± 4 | 33 ± 25 | 31 ± 13 | 12 ± 3 | 9 ± 6 | | B220 | 26 ± 10 | 49 ± 4 | 8 ± 1 | 5 ± 3 | | F4/80 | 1 ± 1 | 8 ± 6 | 43 ± 25 | 27 ± 5 | | GR-1 | 2 ± 1 | 24 ± 18 | 26 ± 1 | 22 ± 4 | |  |  |  |  |  |   Data are the mean (SEM) from two experiments with 3 mice each.  Table S2. Morphological analysis of distinct cell regions (R1-R4) based on size and granularity in the peritoneal cavity 4 days after thioglycollate stimulation. | | | | |
| --- | --- | --- | --- | --- |
| Cell subtype | Regions (%) | | | |
|  | R1 | R2 | R3 | R4 |
| Lymphocytes | 92 ± 3 | 94 ± 3 | 1 ± 0 | 0 ± 0 |
| Macrophages | 7 ± 4 | 5 ± 3 | 97 ± 1 | 6 ± 3 |
| Granulocytes | 0 ± 0 | 1 ± 1 | 1 ± 1 | 94 ± 3 |

Note: Approximately 1,000 cells were counted from each group from each regions defined by size and granularity. All experiments were performed independently and repeated at least three times.
