# Supplementary figures and images for: Selecting the right gate to identify relevant cells for your assay: a study of thioglycollate-elicited peritoneal exudate cells in mice
Source: BMC Res Notes. 2017 Dec 6;10:695. doi: 10.1186/s13104-017-3019-5 (PMC5718147; doi:10.1186/s13104-017-3019-5)

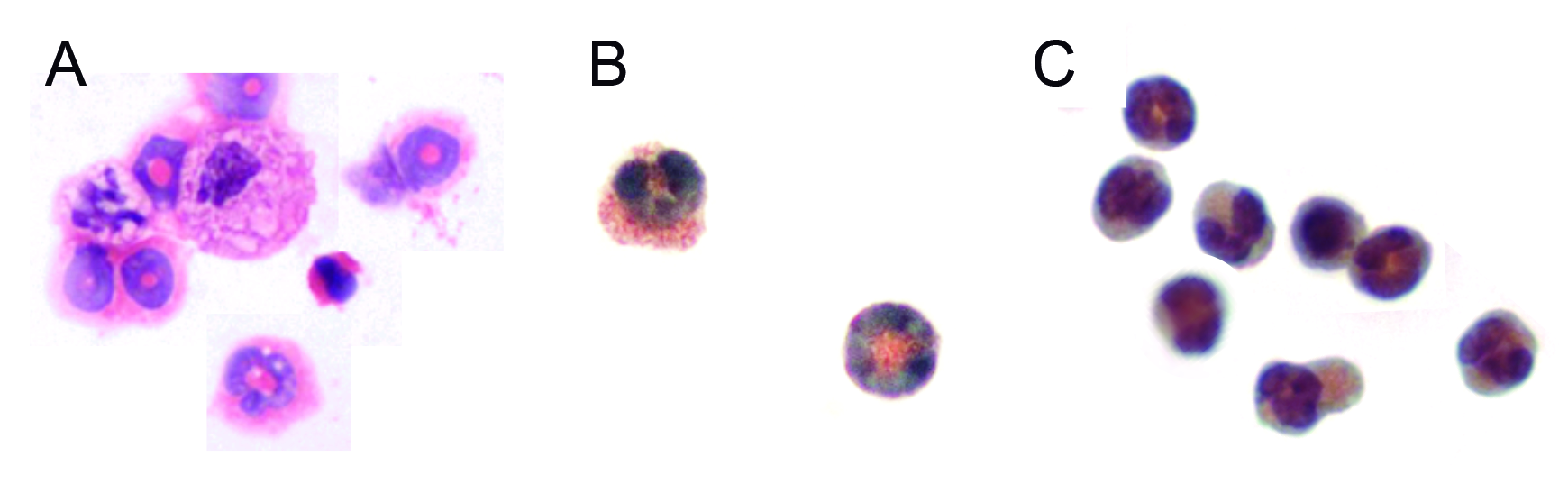

Supplement: Supplementary file 2 — Additional file 2. Sorted cells in the R4 region have eosinophilic cytoplasmic granules. Cells stained with (A) H&E, (B) the Luna method, or (C) the Papanicolau technique. [file 13104_2017_3019_MOESM2_ESM.tif]

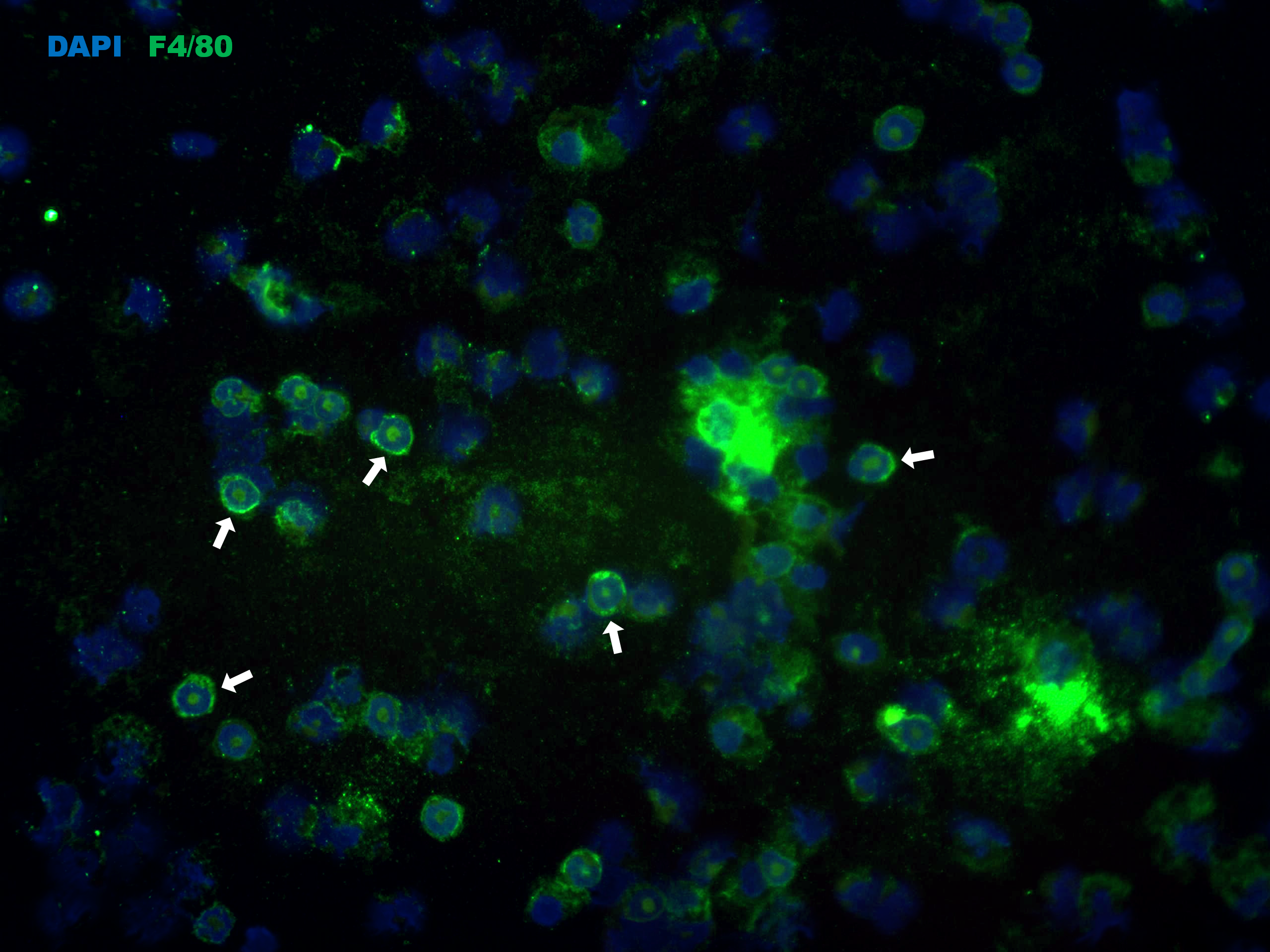

Supplement: Supplementary file 3 — Additional file 3. Cell immunophenotyping: To identify the neutrophils expressed F4/80 antigens, R4 cell from inflammatory PEC were sorted and cytocentrifugated at 500 rpm onto glass slides and fixed in cold acetone and subjected to immunolabeling. The slides were incubated with phosphate buffer saline (PBS) containing 1% bovine serum albumin (BSA), 10% normal goat serum, to block non-specific interactions. Purified F4/80 antigen antibody (Cl:A3-1, catalog # MCAP497, Serotec) was used at 5 µg/ml and incubated for 1 h at room temperature. After, wash the slides with PBS 1× and 0.05% tween 20 and incubated with secondary antibody anti-Rat IgG FITC (catalog # STAR69, Serotec) for 45 min in room temperature. For lobulated nucleus neutrophil identification, the slides were stain with DAPI. [file 13104_2017_3019_MOESM3_ESM.tif]
